# Recent Clinical Studies on the Effects of Tumor Necrosis Factor-Alpha (TNF-α) and Janus Kinase/Signal Transducers and Activators of Transcription (JAK/STAT) Antibody Therapies in Refractory Cutaneous Sarcoidosis: A Systematic Review

**DOI:** 10.7759/cureus.44901

**Published:** 2023-09-08

**Authors:** Stacy L Toriola, Travis Satnarine, Zareen Zohara, Ademiniyi Adelekun, Kofi D Seffah, Korlos Salib, Lana Dardari, Maher Taha, Purva Dahat, Sai Sri Penumetcha

**Affiliations:** 1 Pathology, California Institute of Behavioral Neurosciences and Psychology, Fairfield, USA; 2 Medicine, St. George's University School of Medicine, New York, USA; 3 Pediatrics, California Institute of Behavioral Neurosciences and Psychology, Fairfield, USA; 4 Internal Medicine, California Institute of Behavioral Neurosciences and Psychology, Fairfield, USA; 5 Family Medicine, California Institute of Behavioral Neurosciences and Psychology, Fairfield, USA; 6 Internal Medicine, Piedmont Athens Regional Medical Center, Athens, USA; 7 General Practice, El-Demerdash Hospital, Cairo, EGY; 8 Medical Student, St. Martinus University, Willemstad, CUW; 9 General Medicine, California Institute of Behavioral Neurosciences and Psychology, Fairfield, USA; 10 General Medicine, Chalmeda Anand Rao Institute of Medical Sciences, Telangana, IND

**Keywords:** cutaneous sarcoidosis, -dermatopathology, jak-stat inhibitor, non-caseating granuloma, refractory sarcoidosis, tnf alpha inhibitor

## Abstract

The widely accepted standard of care for chronic cutaneous sarcoidosis is corticosteroids. However, when this treatment is shown to be refractory, other interventions must be considered. In this review, we report the current progress of clinical studies on various monoclonal antibody therapies and their future potential as primary interventions for refractory cutaneous sarcoidosis. In this systematic review, clinical studies on the management of refractory cutaneous sarcoidosis were retrieved from PubMed and ScienceDirect databases. Studies were screened based on article type, publication within the last 10 years, and access to free full text. The articles selected consisted of case studies, clinical trials, and observational studies. The studies needed to focus on cases of diagnosed cutaneous sarcoidosis at the time of the study and involve adult patients resistant to corticosteroid regimens, with or without additional immunomodulators. Only interventions that included tumor necrosis factor-alpha (TNF-α) (e.g., infliximab and adalimumab) or Janus kinase/signal transducers and activators of transcription (JAK/STAT) (e.g., ruxolitinib and tofacitinib) antibody therapy were considered. Two authors independently conducted quality assessments using the Joanna Briggs Institute Critical Appraisal and NIH Study Quality Assessment tools. A total of 16 clinical studies were included in this systematic review using the Preferred Reporting Items for Systematic Reviews and Meta-Analyses (PRISMA) flow diagram. Of the 16 cases included, 15 studies demonstrated partial to complete resolution of cutaneous lesions within a range of two weeks to 18 months from initiation of antibody therapy. Studies on anti-TNF-α intervention demonstrated the most adverse events, including two deaths and one case associated with cutaneous exacerbation. Studies on anti-JAK-STAT interventions demonstrate no adverse events after treatment; however, patient study size was limiting. Recent studies have shown promising potential for anti-TNF-α and anti-JAK-STAT inhibitors to become the mainstay interventions in refractory cutaneous sarcoidosis. Due to limited population studies, the current data on the efficacy and safety of antibody therapies have not yielded a standardized FDA-approved steroid-sparing treatment. Therefore, a need for more population studies on the effectiveness of third-line intervention in refractory cutaneous sarcoidosis is necessary.

## Introduction and background

Sarcoidosis is a chronic multisystem disorder characterized by noncaseating granulomatous inflammation [1]. The highest cases of sarcoidosis reported in the United States are predominately female patients and patients of African-American ancestry, with pulmonary disease as the most common manifestation [2]. The current incidence and prevalence of sarcoidosis are 7.6-8.4 and 59.0-60.1 per 100,000 persons, respectively, while the worldwide incidence and prevalence vary based on ethnicity and geographical location [2,3,4]. Cutaneous sarcoidosis (CS), a subtype of sarcoidosis, is among the most common extrapulmonary manifestations of sarcoidosis. CS manifests in 25-35% of all diagnosed cases of sarcoidosis in the United States [5]. The dermatological presentation of CS is highly variable and can manifest as an isolated condition or in the early to late stages of the systemic sarcoidosis form [5,6]. Because of the differences in CS clinical presentation, it is essential to identify the cutaneous lesion for early diagnosis and treatment [5]. The first-line therapy for CS is corticosteroids [7]. Corticosteroids can be supplemented with second-line therapies such as methotrexate, hydroxychloroquine, leflunomide, azathioprine, mycophenolate, or cyclophosphamide to maximize clinical response [7,8]. Because of the systemic effect of first-line and second-line therapies on the body, there is a greater risk of side effects when used in long-term management [8]. In addition, up to 30% of patients who develop chronic CS may develop resistance to corticosteroids and second-line therapies [9,10]. Recent clinical studies have shown the potential effectiveness of third-line therapies that target the tumor necrosis factor-alpha (TNF-α) cytokine and the Janus kinase/signal transducers and activators of transcription (JAK/STAT) receptor. The preferred management of refractory CS is shifting towards more localized and target-specific interventions. This systematic review aims to analyze and report the most recent clinical outcomes for adult patients with refractory CS who were treated with third-line interventions, such as TNF-α or JAK-STAT antibody therapy.

## Review

Method

This systematic review gathered and screened information using Preferred Reporting Items for Systematic Reviews and Meta-Analyses (PRISMA) guidelines from PubMed and ScienceDirect databases. Articles were searched based on a keyword mesh for the initial research strategy. Criteria for including clinical studies in the review were based on a set timeline, article type, and the availability of free full texts from the databases. In our exclusion process, we screened articles using the keywords "adalimumab and cutaneous sarcoidosis," "infliximab and cutaneous sarcoidosis," "tofacitinib and cutaneous sarcoidosis," and "ruxolitinib and cutaneous sarcoidosis." Articles were excluded if they did not fall within a 10-year publication period from 2013 to 2023, provided free full access to text, and consisted of case reports, case series, randomized or non-randomized clinical trials, or observational studies. Selected articles were organized using ENDNOTE software, and duplicate articles were removed manually. The inclusion criteria for this review required studies that consisted primarily of adult patients, confirmed diagnosis for chronic CS, reported history of resistance to corticosteroid or secondary immunomodulator, and patients receiving either anti-TNF-α (infliximab and adalimumab) or anti-JAK-STAT (tofacitinib and ruxolitinib) antibody therapy. Clinical outcomes for therapeutic interventions in the studies were reported based on changes in physical exam findings, the timeline of improvement from the initial treatment, pre-post diagnostic analysis, and scaled using the Cutaneous Sarcoidosis Activity and Morphology Instrument (CSAMI) assessment tool. Two authors independently assessed the quality of the research studies selected for this review using the Joanna Briggs Institute Critical Appraisal and NIH Quality Assessment tools.

Result

**Figure 1:**
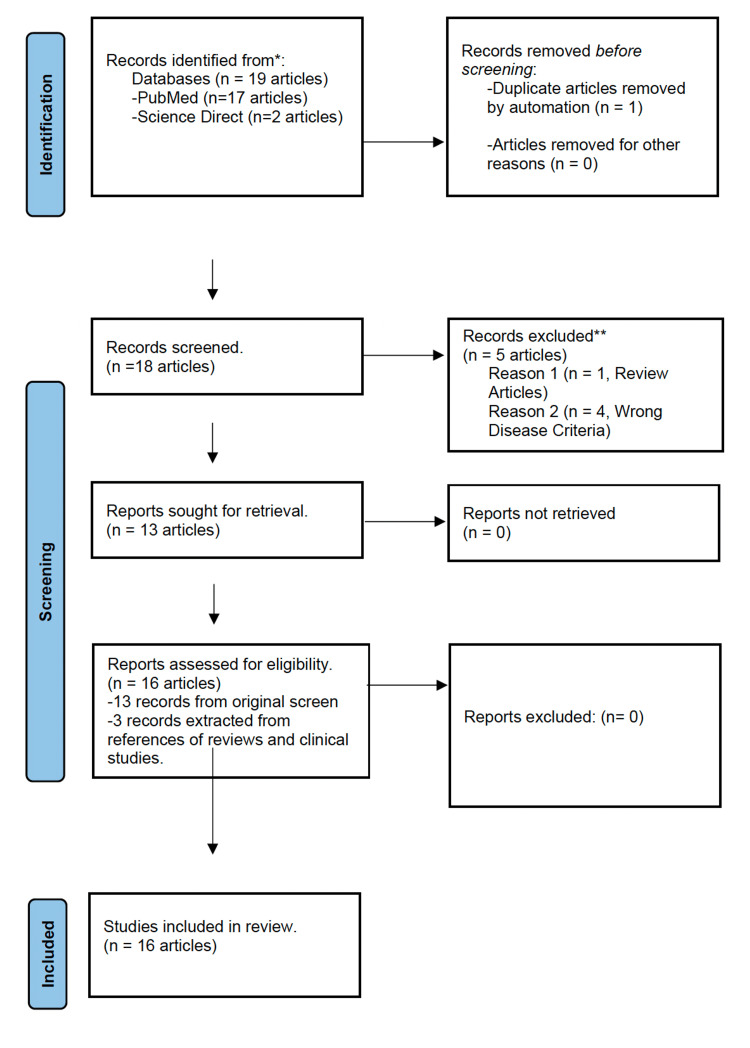
PRISMA flow diagram for systematic review. PRISMA: Preferred Reporting Items for Systematic Reviews and Meta-Analyses.

Using the PRISMA guidelines, we initially screened 19 articles. Six were removed based on the inclusion and exclusion criteria (Figure 1) [11]. Three original articles cited in a past published review on sarcoidosis were identified, that met the inclusion criteria, and were then included in this systematic review. Thus, a total of 16 articles, comprising case reports, open-label clinical trials, and observational (both prospective and retrospective) studies, were included. Quality checks performed on all studies indicated a low to moderate risk of bias (Tables 1-2) [12-28].

**Table 1:** National Heart, Lung, and Blood Institute: Study Quality Assessment Tool. The table presents findings from the Study Quality Assessment Tool created by the National Heart, Lung, and Blood Institute. This assessment tool was utilized to evaluate the quality of pre-post observational study designs included in this review [12].

Quality Assessment Tool for Before-After (Pre-Post) Studies With No Control Group Criteria: Yes/No	Sakkat A et al. (2022) [14]	Heidelberger V et al. (2017) [15]	Damsky W et al. (2019) [24]	Damsky W et al. (2022) [25]
Was the study question or objective clearly stated?	Yes	Yes	Yes	Yes
Were eligibility/selection criteria for the study population prespecified and clearly described?	Yes	Yes	Yes	Yes
Were the participants in the study representative of those who would be eligible for the test/service/intervention in the general or clinical population of interest?	Yes	Yes	Yes	Yes
Were all eligible participants that met the prespecified entry criteria enrolled?	Yes	Yes	Yes	Yes
Was the sample size sufficiently large to provide confidence in the findings?	Yes	Yes	No	Yes
Was the test/service/intervention clearly described and delivered consistently across the study population?	Yes	Yes	Yes	Yes
Were the outcome measures prespecified, clearly defined, valid, reliable, and assessed consistently across all study participants?	Yes	Yes	Yes	Yes
Were the people assessing the outcomes blinded to the participants' exposures/interventions?	No	No	No	No
Was the loss to follow-up after baseline 20% or less?	No	No	Yes	No
Were those lost to follow-up accounted for in the analysis?	No	No	Yes	No
Did the statistical methods examine changes in outcome measures from before to after the intervention?	Yes	Yes	Yes	Yes
Were statistical tests done that provided p values for the pre-to-post changes?	Yes	Yes	Yes	Yes
Were outcome measures of interest taken multiple times before the intervention and multiple times after the intervention (i.e., did they use an interrupted time-series design)?	Yes	Yes	Yes	Yes
If the intervention was conducted at a group level (e.g., a whole hospital, a community, etc.) did the statistical analysis take into account the use of individual-level data to determine effects at the group level?	No	No	No	No
Quality Rating: Good, Fair, or Poor	Good	Good	Good	Good

**Table 2:** Critical appraisal tool for case reports. The table presents findings from the critical appraisal assessment tool developed by the Joanna Briggs Institute. This tool was used to evaluate the quality of case reports included in this review [13].

Case Series criteria: Yes, No, Unclear, Not Applicable	Hagan CE et al. (2018) [18]	Mirzaei A et al. (2017) [9]	Sussman ME et al. (2021) [16]	Vetos D et al. (2021) [17]	Santos G et al. (2013) [19]	Wei JJ et al. (2019) [21]	Rotenberg C et al. (2018) [20]	Talty R et al. (2021) [22]	Singh K et al. (2020) [23]	Damsky W et al. (2018) [26]	Damsky W et al. (2020) [27]	Alam M et al. (2020) [28]
Were the patient’s demographic characteristics clearly described?	Yes	Yes	Yes	Yes	Yes	Yes	Yes	No	Yes	Yes	Yes	Yes
Was the patient’s history clearly described and presented as a timeline?	Yes	Yes	Yes	Yes	Yes	Yes	Yes	No	Yes	Yes	Yes	Yes
Was the current clinical condition of the patient in the presentation clearly described?	Yes	Yes	Yes	Yes	Yes	Yes	Yes	No	Yes	Yes	Yes	Yes
Were diagnostic tests or assessment methods and the results clearly described?	Yes	Yes	Yes	Yes	Yes	Yes	Yes	No	Yes	Yes	Yes	Yes
Was the intervention(s) or treatment procedure(s) clearly described?	Yes	Yes	Yes	Yes	Yes	Yes	Yes	No	Yes	Yes	Yes	Yes
Was the post-intervention clinical condition clearly described?	Yes	Yes	Yes	Yes	Yes	Yes	Yes	No	Yes	Yes	Yes	Yes
Were adverse events (harms) or unanticipated events identified and described?	No	No	No	No	Yes	No	No	No	No	No	No	No
Does the case report provide takeaway lessons?	Yes	Yes	Yes	Yes	Yes	Yes	Yes	Yes	Yes	Yes	Yes	Yes
Overall Appraisal: Include, Exclude, Seek Further Info	Include	Include	Include	Include	Include	Include	Include	Include	Include	Include	Include	Include

**Figure 2:**
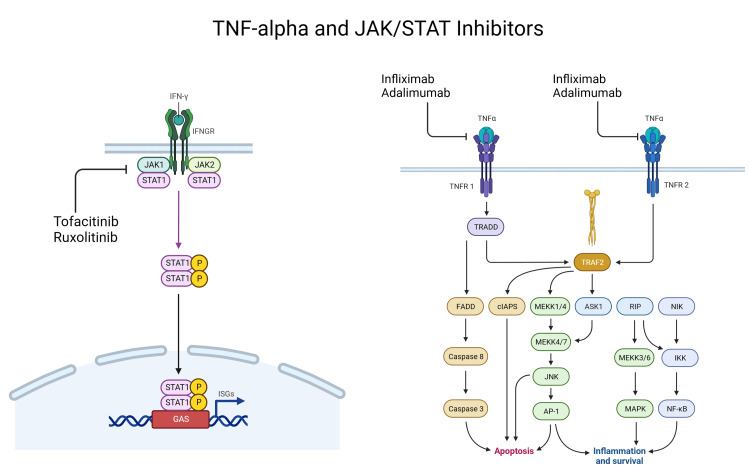
The mechanism of TNF-α and JAK-STAT inhibitors. The binding of interferon-gamma (INF-γ) ligand to the INF-γ receptor activates the JAK-STAT signaling cascade. On the left, tofacitinib and ruxolitinib inhibit the JAK-STAT complex, thereby halting the pro-inflammatory signaling cascade. On the right, infliximab and adalimumab work to prevent the activation of the TNF-α ligand binding to the TNF-α receptor, thereby interrupting downstream cellular functions. The illustration and publication license were provided by BioRender.com. TNF-α: Tumor Necrosis Factor-alpha; JAK-STAT: Janus kinase/Signalling transducer of activators of transcription.

In this review, our study populations included adult patients with either CS alone or as a manifestation of systemic sarcoidosis. Patients were treated with either anti-TNF-α (infliximab or adalimumab) or anti-JAK-STAT (tofacitinib or ruxolitinib) inhibitors. These treatments were administered either as stand-alone therapies or in combination with corticosteroids and second-line immunomodulators. In some cases, they were introduced as alternate therapies when corticosteroids and second-line immunomodulators failed to clear cutaneous lesions. The mechanisms of action for anti-TNF-α and anti-JAK-STAT therapies are depicted in Figure 2. Detailed descriptions of the antibody therapy interventions and the associated clinical outcomes can be found in Table 3.

**Table 3:** Summary of clinical outcomes for refractory cutaneous sarcoidosis treated with TNF-α and JAK/STAT inhibitors. TNF-α: Tumor Necrosis Factor-alpha (infliximab and adalimumab); JAK-STAT: Janus kinase/Signalling transducer of activators of transcription (ruxolitinib and tofacitinib); CS: Cutaneous sarcoidosis; CSAMI: Cutaneous Sarcoidosis Activity and Morphology Instrument.

Author & Year	Study Type	Antibody Therapy	Additional Therapy	Study Size	Therapeutic Response Reported
Sakkat A et al. (2022) [14]	Retrospective	Infliximab	Prednisone	15	91.7% complete reduction in CS lesions
Heidelberger V et al. (2017) [15]	Retrospective and Prospective	Infliximab	Prednisone	46	79% complete resolution after 12 months
Hagan CE et al. (2018) [18]	Case Report	Adalimumab		1	Complete resolution after 4 months
Mirzaei A et al. (2017) [9]	Case Report	Adalimumab	Prednisolone	1	Complete resolution after 2 weeks
Sussman ME et al. (2021) [16]	Case Report	Adalimumab		1	Partial Resolution after 9 months
Vetos D et al. (2021) [17]	Case Report	Adalimumab	Pentoxifylline	1	Partial Resolution after 8 months
Santos G et al. (2013) [19]	Case Report	Adalimumab	Methotrexate	1	CS lesion exacerbation after 3^rd^ dose.
Wei JJ et al. (2019) [21]	Case Report	Ruxolitinib		1	Complete resolution in 5 months
Rotenberg C et al. (2018) [20]	Case Report	Ruxolitinib	Prednisone	1	Complete resolution in 3 months
Talty R et al. (2021) [22]	Case Report	Tofacitinib		1	Complete resolution in 6 months
Singh K et al. (2020) [23]	Case Report	Tofacitinib	Pulsed Dye Laser Therapy	1	Partial resolution after 10 weeks
Damsky W et al. (2019) [24]	Prospective	Tofacitinib		3	96% CSAMI score improvement after 4 months
Damsky W et al. (2022) [25]	Open Label Clinical Trial	Tofacitinib		10	82.7% CSAMI score improvement after 6
Damsky W et al. (2018) [26]	Case Report	Tofacitinib		1	CSAMI score improvement from 85 to 0 in 10 months
Damsky W et al. (2020) [27]	Case Report	Tofacitinib		1	Complete resolution after 4 months
Alam M et al. (2020) [28]	Case Report	Tofacitinib	0.1 % topical triamcinolone acetonide and extra virgin olive oil	1	CSAMI score improvement from 9 to a 5 in 5 months

Infliximab is an anti-TNF-α monoclonal antibody therapy that has shown the most efficacy as a third-line intervention in recent years. Two studies among those reviewed reported on the clinical outcomes of infliximab therapy in patients with refractory CS. The studies included one retrospective and one retrospective-prospective observation, with no control group in either study. In the study by Sakkat A et al., 33 patients with refractory systemic sarcoidosis were treated with infliximab and prednisone. Of the 33 patients, 15 were identified with refractory CS and received an infliximab regimen of 3-5 mg/kg at 0, 2, and 6 weeks. The infliximab regimen was prolonged for four to six weeks in patients with varied clinical responses. By the end of the study, there was a reduction in prednisone need by 50% in the total patient population. Of the 15 patients with refractory CS, there was a 91.7% resolution of CS lesions [14]. Seventeen patients reported adverse events, and one patient was reported to have passed away from respiratory failure. The adverse events included pneumonia, leukopenia, infusion reaction, minor infections, paresthesia, anaphylaxis, lesion exacerbation, chest pain, headache, and asthma. Seven patients were required to discontinue therapy due to these adverse events [14]. In the study by Heidelberger V et al., 46 of the 140 patients were positively identified with refractory CS. Patients were either given infliximab or infliximab with corticosteroid and a second immunomodulator. The clinical outcomes were measured based on an overall complete cutaneous response rate. The patient demonstrated a complete cutaneous response rate of 24% at three months, 46% at six months, and 79% at 12 months [15]. A total of 14 of the 46 patients experience infectious adverse events of varying severity. In the overall study, half of the patient population relapsed after discontinuation of therapy [15]. Patients who resumed therapy after relapse were able to achieve remission [15]. Similarly, adalimumab is also an anti-TNF-α monoclonal antibody therapy that has yet to be widely researched in refractory CS. Four of the 16 clinical studies included in this review reported the efficacy of adalimumab use in refractory CS. All four studies were case reports and consisted of patients administered with the adalimumab regimen of 40 mg injected subcutaneously every two weeks and oral corticosteroids with or without additional second-line immunomodulators. In the study by Sussman ME et al., only one of the two patients studied required adalimumab therapy. After nine months, the patient began to show partial resolution of CS lesions with complete remission after 21 months [16]. Another case study by Mirzaei A et al. in 2017 demonstrated complete CS lesion resolution two weeks after the first dose was administered and discontinuation of prednisone after three months [9]. In case studies by Vetos D et al. and Hagan CE et al., both patients experience complete resolution after four months of therapy [17, 18]. In three of the four case reports, no adverse events were observed in the patient treated [9, 17, 18]. However, one case reported that adalimumab intervention triggered CS exacerbation. In the study by Santos G et al. in 2013, the patient received a combination regimen of isoniazid (300 mg/day), methotrexate (7.5 mg/wk), and methylprednisolone (4 mg/day [19]. Adalimumab was administered two months after the initial isoniazid dose. After the third dose of adalimumab was given, the patient began to exhibit cutaneous exacerbation, but complete resolution of the lesion occurred shortly after adalimumab had been discontinued [19]. Two case studies reported on the clinical outcomes of ruxolitinib therapy in refractory CS. In the study by Rotenberg C et al., ruxolitinib was initiated in a patient to treat malignant polycythemia vera, who also had a comorbidity with cutaneous sarcoidosis. The patient received 5 mg of ruxolitinib every 12 hours. The patient demonstrated complete resolution of CS lesions after three months and tapering off steroids safely after six months [20]. Conversely, in the Wei study, a patient received 10 mg of ruxolitinib twice daily. This patient achieved complete lesion resolution after five months [21]. Notably, neither case study reported any adverse events. Seven studies, encompassing case reports, open-label clinical trials, and observational studies, detailed clinical outcomes of tofacitinib therapy in refractory CS. In these studies, the tofacitinib regimen consisted of either 2% topical ointment, a 5 mg oral dose twice daily with or without corticosteroid, and a second line of immunomodulators. In six of the seven studies, complete resolution of CS lesions was reported within two and a half months to 10 months [22-28]. In 2022, the study by Damsky W et al. showed 82.7% resolution of the CS lesion in 10 patients after tofacitinib therapy [25]. One case highlighted a patient who, although lost to follow-up, showed complete resolution after initial therapy [27]. In the case study by Singh K et al., the resolution of CS lesions was reported after ten weeks of therapy. However, an additional three months of pulse dye laser therapy was required to treat the patient's persistent telangiectasia [23]. Notably, none of the reviewed studies reported any adverse events.

Discussion

The diagnosis of CS requires a punch or incisional wedge biopsy [5]. A positive CS biopsy would reveal infiltration of non-caseating granuloma, epithelioid cells, and lymphocytes into the surrounding epithelia tissue [5, 29]. A study by Rosenbach M et al. established an assessment tool to quantify the severity of CS lesions using the Cutaneous Sarcoidosis Activity and Morphology Instrument (CSAMI) [30]. The CSAMI tool can measure the therapeutic response in CS treatments. The appearance of CS lesions can be distinguished based on color, size, and contour and varies based on the body's location and the patient's skin complexion [6]. CS lesions are classified into non-specific or specific variants, but the specific variants exclusively demonstrate the diagnostic non-caseating granulomatous feature on biopsy [5]. Amongst the specific variant types, papules and plaques present with the highest frequency in patients [31]. Other specific variants of CS lesions include lupus pernio, scar sarcoidosis, subcutaneous nodules, hypopigmented macules, psoriasiform, ulcerative, erythrodermic, ichthyosiform, and lesions of the scalp and nails [6]. Studies on the variability in CS presentation among multiethnic patient populations have yet to be well-defined. For example, the color of papule and plaque lesions vary across the Fitzpatrick skin type spectrum. These lesions can appear light brown, red, or violaceous for patients with lighter skin complexion [31]. In comparison, the same lesion type will appear as dark brown, purple, or burgundy in patients with more melanin in their complexion [6, 31]. The frequency of CS lesion type has also been shown to be influenced by ethnicity. There is a higher frequency of subcutaneous nodule forms in patients of European ancestry compared to patients of African ancestry, who show a higher frequency of psoriasiform and hypopigmented macule forms [6].

**Figure 3:**
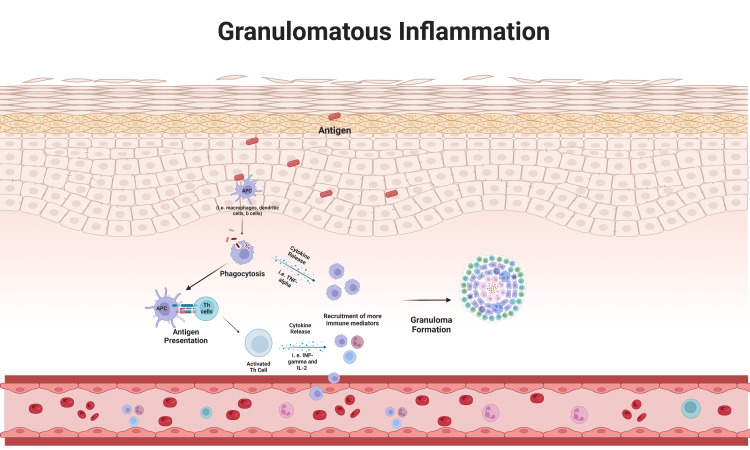
Granulomatous inflammation. In granulomatous inflammation, antigens are recognized and phagocytosed by antigen-presenting cells (APCs) such as macrophages, dendritic cells, and B lymphocytes. After the antigen is metabolized, the APC can activate naïve T helper lymphocytes through MHC II-mediated interactions. Both the APC and T helper lymphocytes can promote the recruitment of inflammatory mediators to the inflammation site by releasing pro-inflammatory cytokines, including Tumor Necrosis Factor alpha, Interferon gamma, and Interleukin-2. This ongoing process results in the sequestration of antigens by clusters of macrophages and lymphocytes, forming a granuloma. The illustration and publication license were provided by BioRender.com. MHC II: Major Histocompatibility Complex II.

Varying triggered events can cause the chronic inflammatory condition seen in CS, but the primary cause has not been well defined. Over the decades, scientists have argued that environmental and genetic factors may contribute to the development of CS [1, 2, 4, 7]. These risk factors include infectious pathogens, inorganic and organic agents, metals, medication, and genetic polymorphisms [1]. The granulomatous inflammatory response observed in CS has been linked to T lymphocyte-mediated response to environmental and genetic triggering factors. The activation of T lymphocytes by cytokine (i.e., TNF-α) released from antigen-presenting cells, like macrophages and dendritic cells, leads to the differentiation of T lymphocytes and the release of more specialized proinflammatory cytokines (i.e., interleukin-2 and interferon-Gamma). The release of proinflammatory cytokines triggers a cascade of events, including the recruitment of more cellular inflammatory mediators and further potentiation of the inflammatory response [32]. A past study on the pathology of sarcoidosis demonstrated that the disturbance of the regulatory T lymphocyte subtype, Th-17, triggers an inflammatory cascade involving the activation of both T and B lymphocytes and the production of disease-causing autoantibodies [32, 33]. Because of the varying triggered events that can induce CS, it is unclear whether T and B lymphocytes undergo activation solely by antigen presentation activation, dysregulated autoimmunologic means, or both. However, it is understood that the inability of the immune system to neutralize the offending antigen or sequestration of autoinflammatory response eventually leads to the migration and differentiation of macrophages to epithelioid cells, thus initiating the granulomatous process [32, 34, 35]. Figure 3 illustrates the mechanism of granuloma formation.

Genetic predisposition plays an essential role in symptom variability and clinical response to treatment. Several key findings should be noted when examining multiethnic populations with variability seen in the clinical presentation of sarcoidosis. Past studies have identified that patients of African-American ancestry are commonly diagnosed about 10 years earlier than patients of European-American ancestry due to the early onset of symptoms [2, 36]. Studies have also shown that patients of African-American ancestry with the same stage of the disease as any other multiethnic group will display higher granuloma density, which may be associated with a genetic predisposition to a more severe presentation of sarcoidosis [2, 37]. The human leukocyte antigen (HLA) is a system of genes that have been shown to predispose patients to cutaneous sarcoidosis and its systemic form [32, 38]. The HLA antigen genes produce protein complexes that regulate the human immune system. The genetic predisposition associated with HLA antigen has been shown to influence variability seen in sarcoidosis. Chronic sarcoidosis, sarcoidosis with extrapulmonary symptoms, and Lofgren syndrome have all been linked to patients' carriers of the Class II Genotype HLA DRRB1 [32, 38, 39, 40]. In particular, the Lofgren syndrome phenotype is more frequent among females with sarcoidosis. It can present acutely with various symptoms, like fever, bilateral hilar lymphadenopathy, lower extremity arthritis, and erythema nodosum (a non-specific variant) [41-43]. In the US, patients of African and European descent are statistically the most affected by sarcoidosis [2]. Both patient populations have shown links between sarcoidosis and the HLA DRB1*11:01 genotype [32]. More specifically, patients of European descent have been linked to HLA DRB1 15:01, HLA DRB1 4:0, and HLA DRB1*3:01 genotype susceptibility, whereas patients of African descent have been linked to HLA DRB1* 12:01, DRB1*15:03, and DQB1 0602 genotype susceptibility [32, 39, 42, 44, 45]. Interestingly, the HLA DRB1*3:01 genotype demonstrates an increased susceptibility to sarcoidosis in European descent and provides protective regulatory effects against sarcoidosis in patients of African descent [32, 45].

The prognosis of CS can also vary among patients. Mild presentations of CS can resolve spontaneously. In a more moderate-to-severe case, corticosteroid monotherapy is the standard of care. However, long-term use of corticosteroids has been linked to various comorbidities and can significantly hinder the patient's quality of life. These adverse effects include weight gain, hypertension, behavioral changes, diabetes, osteoporosis, recurrent infections, and worsening of pre-existing comorbidities [7, 46, 47, 48]. Corticosteroid intervention can also become refractory in some patient populations with CS. In this case, second and third-line immunomodulators are supplemented with corticosteroid therapy or used as a monotherapy. Population studies on the efficacy and safety of second and third-line interventions are limited and report less efficacy evidence than corticosteroid therapy [7]. This review focuses on the efficacy studies and evidence in anti-TNF-α and anti-JAK-STAT therapeutic interventions. These interventions are without risk, but recent studies have shown that some third-line alternatives may become a first-line option for refractory CS than corticosteroids alone. TNF-α is a cytokine that is released during proinflammatory responses. It can assist in several cellular cascades, including aging, infectious response, tumor suppression, and cell death [8]. In CS, TNF-α is upregulated because of the overactive macrophage population, which mediates granulomatous formation [8, 49]. The genes that transcribe TNF-α consist of polymorphisms that play a role in the variability seen in therapeutic interventions. TNF-α G-308A polymorphism has been shown to influence clinical outcomes in sarcoidosis patients treated with infliximab and adalimumab [50]. In this review, we reported six studies on the anti-TNF-α intervention that resulted in partial to complete resolution of CS lesions after several months. Interventions included infliximab or adalimumab as a monotherapy or combination with corticosteroid and a second immunomodulator. In both infliximab studies, there were reports of adverse events and deaths due to medical complications. Comparatively, most adalimumab studies demonstrated complete resolution of CS lesions with no adverse events. 

One study reviewed adalimumab interventions that demonstrated CS exacerbation. The combination regimen this patient was on while receiving adalimumab differed from the therapeutic interventions seen in all the other adalimumab studies. Genetic predisposition and medical cross-reactions should be taken into account when comparing the results of this intervention to similar studies. Additionally, the study population size for infliximab and adalimumab was limited, making it challenging to demonstrate significant efficacy and safety evidence for the treatment of refractory CS. These limitations underscore the need for further research in this area of study. Similar to the TNF-α pathway, the JAK-STAT pathway is a cell signaling cascade that the immune system commonly regulates, particularly during interferon-gamma-mediated inflammatory responses. Recent studies on refractory CS indicate that JAK-STAT inhibitors, such as tofacitinib and ruxolitinib, can offer a more localized intervention, reducing the risk of adverse events observed with other third-line interventions. In this review, we highlight ten studies that show partial to complete resolution of refractory CS lesions using these therapies. The genes that regulate the JAK-STAT pathway demonstrate genetic polymorphism that can influence variability in the clinical response of the study population. The majority of patients experience complete resolution of CS lesions within a year of initiating treatment, but the time of response greatly varied. Although the sample size review was small, none of the studies reported adverse events in the patients treated. The JAK-STAT inhibitors could be a superior intervention for refractory CS. However, larger study populations will be needed to address this question.

Limitations

Many cases reviewed demonstrated the potential for anti-TNF-α and anti-JAK-STAT inhibitors to become the mainstay monotherapy for refractory CS. Our exclusion and inclusion criteria for articles included in our review were limited, particularly those with free open-access articles only. In addition, several cases were case reports, as larger sample sizes for case series, clinical trials, and observational studies were limited.

## Conclusions

Third-line modulators must be considered when corticosteroids and second-line therapies are insufficient in keeping cutaneous sarcoidosis in remission. This review reported on the current clinical outcomes of refractory CS interventions. Due to limited population studies, the data on the efficacy and safety of steroid-sparing agents have yet to yield a more standardized alternative for refractory CS therapy. However, recent studies have shown promising potential for anti-TNF-α and anti-JAK-STAT inhibitors to become the mainstay monotherapy. However, in some studies, anti-TNF-α inhibitors demonstrated a variety of adverse events, including one cutaneous exacerbation. On the other hand, anti-JAK-STAT inhibitors show more consistent clinical outcomes with complete remission, reduction in steroid need, and no reported adverse effects. However, the sample size for these studies needs to be improved. Therefore, more extensive population studies are needed on the effectiveness of third-line intervention for moderate to severe CS.
