# Correction: Recent Clinical Studies on the Effects of Tumor Necrosis Factor-Alpha (TNF-α) and Janus Kinase/Signal Transducers and Activators of Transcription (JAK/STAT) Antibody Therapies in Refractory Cutaneous Sarcoidosis: A Systematic Review

**DOI:** 10.7759/cureus.c343

**Published:** 2025-10-03

**Authors:** Stacy L Toriola, Travis Satnarine, Zareen Zohara, Ademiniyi Adelekun, Kofi D Seffah, Korlos Salib, Lana Dardari, Maher Taha, Purva Dahat, Sai Sri Penumetcha

**Affiliations:** 1 Pathology, California Institute of Behavioral Neurosciences and Psychology, Fairfield, USA; 2 Medicine, St. George's University School of Medicine, New York, USA; 3 Pediatrics, California Institute of Behavioral Neurosciences and Psychology, Fairfield, USA; 4 Internal Medicine, California Institute of Behavioral Neurosciences and Psychology, Fairfield, USA; 5 Family Medicine, California Institute of Behavioral Neurosciences and Psychology, Fairfield, USA; 6 Internal Medicine, Piedmont Athens Regional Medical Center, Athens, USA; 7 General Practice, El-Demerdash Hospital, Cairo, EGY; 8 Medical Student, St. Martinus University, Willemstad, CUW; 9 General Medicine, California Institute of Behavioral Neurosciences and Psychology, Fairfield, USA; 10 General Medicine, Chalmeda Anand Rao Institute of Medical Sciences, Telangana, IND

This article has been corrected to fix the following typos in the PRISMA diagram:

- 'Reports sought for retrieval' corrected from 12 to 13
- 'Reports excluded' corrected from 5 to 0

